# Intraprostatic Hydatid Cyst: An Unusual Presentation

**DOI:** 10.1100/tsw.2006.360

**Published:** 2006-01-19

**Authors:** Yassine Nouira, Mohamed Y. Binous, Kais Nouira, Amina Mekni, Yousri Kallel, Zouhaier Fitouri, Sataa Sallami, Ali Horchani

**Affiliations:** ^1^ Department of Urology, La Rabta Hospital, Tunis, Tunisia; ^2^ Department of Radiology, La Rabta Hospital, Tunis, Tunisia; ^3 ^ Department of Pathology, La Rabta Hospital, Tunis, Tunisia

**Keywords:** echinococcosis, hydatid cyst, prostate, treatment, endoscopy

## Abstract

A case of intraprostatic cyst is reported. The patient presented with a completely evacuated hydatid cyst of the prostate. The intraprostatic cystic cavity that was communicating with the urethra developed urinary stones. The patient had transurethral resection of the prostate, the stones in the cyst were pushed into the bladder and fragmented using a ballistic lithotripter. Pathological examination concluded to a prostatic hydatid cyst that had evacuated through the urethra and was complicated by stone formation within the residual cavity. Postoperative course was uneventful and follow-up did not show evidence of recurrence. This is the first case of hydatid cyst of the prostate to present as an intraprostatic stone pouch.

## INTRODUCTION

Echinococcosis (or hydatid disease) is a parasitic infestation caused by the development of the larval cystic stage of a cestode *Echinococcus granulosus* (*E. granulosus*). In the parasitic cycle, herbivorous animals and especially sheep are normal intermediate hosts and this explains that hydatidosis is endemic in sheep-raising areas[1,2].

We report a case of an intraprostatic hydatid cyst presenting as a stone pouch. To our knowledge, this is the first case to be reported in the literature.

## CASE REPORT

A 52-year-old patient, with a history of spontaneous stone passage in urine, was hospitalised for dysuria and burning micturition. On questioning, he denied any history of gross haematuria or grape skin–like material evacuated in urine. Physical examination revealed the prostate to be replaced by a palpable mass, suggesting a pouch full of stones. Renal function as well as total blood count and KUB were normal. Plain film centred on the pelvis showed multiple stones in the prostatic area (Fig. 1). On excretory urography, the prostate seemed to be excavated and full of stones. Transrectal ultrasonography revealed

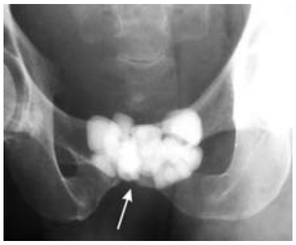

**FIGURE 1.** Plain film centred on the pelvis showing multiple stones in the prostatic area (arrow).

intraprostatic stones with normal seminal vesicles. Chest X-ray as well as abdominal ultrasonography were normal. Urethroscopy showed an intraprostatic cavity containing multiple stones that communicated with the prostatic urethra through multiple orifices of different sizes (Video 1). The stones were pushed back into the bladder before being fragmented with a ballistic lithotripter and evacuated. The patient had transurethral resection of the prostate, allowing wide communication of the bladder with the intraprostatic cystic cavity. Pathological examination of the prostatic specimen showed the cystic cavity to be an intraprostatic hydatid cyst (Fig. 2) evacuated into the prostatic urethra and complicated by stone formation. Postoperative course was uneventful and the patient became asymptomatic.

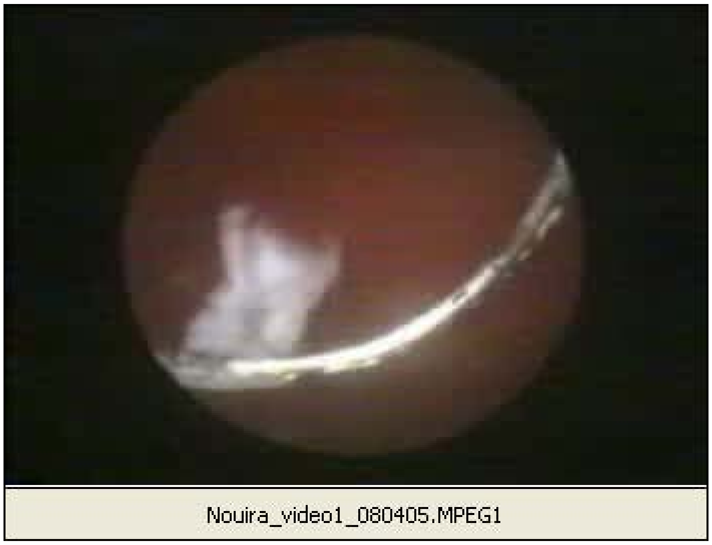

**click here to run video**

## DISCUSSION

Although all organs can be involved by hydatid disease, liver (61%), lung (29%), spleen (5%), and kidneys (4%) account for 99% of hydatid cyst cases[1].

Pathogenesis of the intraprostatic hydatid cyst in the case we report can be explained by haematogenous dissemination with development of the cyst within the prostatic tissue. Erosion of the cystic wall resulted in cystic rupture in the urethral lumen and evacuation of its content.

Hydaturia, which is characterised by the presence in urine of grape skin–like fragments, is the only pathognomonic sign that testifies of a ruptured hydatid cyst in the urinary tract. Unfortunately, this is far away from being constant and can pass undiagnosed, and the case we report is a testimony. Intracystic stone formation is explained by urine stagnation in the remaining cystic cavity.

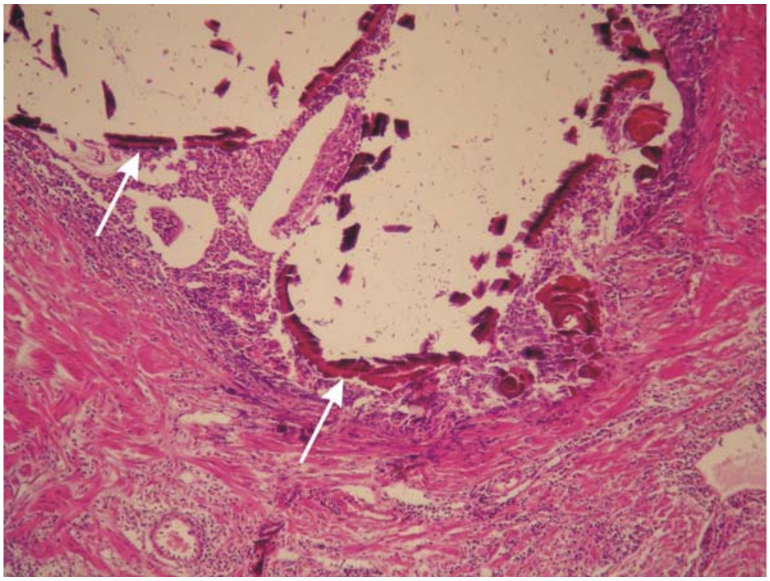

**FIGURE 2.** Photomicrograph of a prostate resection chip showing a calcified hydatid cyst wall (arrow) within the prostatic tissue.

The present case is another demonstration of the diagnostic difficulties of hydatid cyst in the urinary tract. Clinical symptoms are characterised by their paucity as the cyst is slowly growing and remains asymptomatic for a long period of time[1].

Serology tests for hydatid disease are positive in only two-thirds of cases and are of help only when positive[1]. Dead or infected cysts can show negative serology tests[1]. Because hydatid disease was not suspected preoperatively, we did not perform serology tests on the patient. Hydatid cysts can show peripheral curvilinear calcifications on plain films. Ultrasonography can be of valuable help in the diagnosis of hydatid disease. However, in our case, it did not raise suspicion of hydatid cyst because of the unusual localisation and the intracystic stones.

Hydatid cysts of the prostate present as intraprostatic cystic masses[3,4,5,6] and the case we report is the first one to be ruptured in the urethra and complicated with intracystic stones.

Intact cysts can be treated by complete or partial cystic excision after treating the cyst with a scolicidal agent, usually hypertonic saline or hydrogen peroxide. Laparoscopic excision of a hydatid cyst of the prostate was recently reported[3].

In the case we report, the easiness to access the cyst endoscopically rendered endoscopic management with deroofing of the cystic cavity a logical option. Transurethral resection of the prostatic tissue separating the cystic cavity from urethra allowed good drainage with potentially no risk for stone recurrence.

Even though this hydatid localisation was treated, there is need for further follow-up in that additional cysts can appear later in other sites.
